# Blood-derived extracellular vesicles isolated from healthy donors exposed to air pollution modulate in vitro endothelial cells behavior

**DOI:** 10.1038/s41598-020-77097-9

**Published:** 2020-11-18

**Authors:** Federica Rota, Luca Ferrari, Mirjam Hoxha, Chiara Favero, Rita Antonioli, Laura Pergoli, Maria Francesca Greco, Jacopo Mariani, Lorenza Lazzari, Valentina Bollati

**Affiliations:** 1 EPIGET LAB, Department of Clinical Sciences and Community Health, Università Degli Studi Di Milano, Via San Barnaba, 8, 20122 Milan, Italy; 2 Department of Pharmacological and Biomolecular Sciences, Università Degli Studi Di Milano, Milan, Italy; 3 Cell Factory, Laboratory of Regenerative Medicine, Department of Services & Preventive Medicine, Fondazione IRCCS Ca’ Granda Ospedale Maggiore Policlinico, Milano, Italy

**Keywords:** Environmental sciences, Biomarkers, Risk factors

## Abstract

The release of Extracellular Vesicles (EVs) into the bloodstream is positively associated with Particulate Matter (PM) exposure, which is involved in endothelial dysfunction and related to increased risk of cardiovascular disease. Obesity modifies the effects of PM exposure on heart rate variability and markers of inflammation, oxidative stress, and acute phase response. We isolated and characterized plasmatic EVs from six healthy donors and confirmed a positive association with PM exposure. We stratified for Body Mass Index (BMI) and observed an increased release of CD61+ (platelets) and CD105+ (endothelium) derived-EVs after high PM level exposure in Normal Weight subjects (NW) and no significant variations in Overweight subjects (OW). We then investigated the ability to activate endothelial primary cells by plasmatic EVs after both high and low PM exposure. NW-high-PM EVs showed an increased endothelial activation, measured as CD105+/CD62e+ (activated endothelium) EVs ratio. On the contrary, cells treated with OW-high-PM EVs showed reduced endothelial activation. These results suggest the ability of NW plasmatic EVs to communicate to endothelial cells and promote the crosstalk between activated endothelium and peripheral cells. However, this capacity was lost in OW subjects. Our findings contribute to elucidate the role of EVs in endothelial activation after PM exposure.

## Introduction

According to the World Health Organization, air pollution poses a severe risk to cardiovascular (CV) health, with ~ 3% of cardiopulmonary deaths each year being attributable to particulate matter (PM) globally^1^. Acute^2,3^ or chronic^3–5^ PM exposure can trigger the risk of myocardial ischemia, stroke, and arrhythmia, particularly in susceptible populations.

Although the cascade of events linking PM exposure to CV disease development is still largely unknown, increasing evidence has shown that endothelial dysfunction plays a central role in the pathogenesis of CV disease^6^ and it may precede CV events^7^.

The lung represents the first target of inhaled PM, which produces a local inflammatory reaction involving also the pulmonary capillary endothelium^8^. However, PM effect is not only limited to the pulmonary environment, making reliable the existence of a cross talk between the respiratory and CV systems that may underlie the observed peripheral effects of PM exposure^9^. This cross-talk can be exerted through extracellular vesicles (EVs), which are membrane-enclosed vesicles that are released both under physiological and pathological conditions and are able to transfer specific information to other cells^10^. Despite their limited size, EVs play a pivotal role in cell-to-cell communication processes, as they are able to interact with recipient cells transferring a wide range of biologically active molecules, including microRNAs (miRNAs)^11^. As miRNA are involved in post-transcriptional silencing, EVs have attracted increasing interest as possible mediators of cell-to-cell communication^10^. In addition, EVs can modulate the fate of target cells through several biological effectors, which can be part of their content (e.g., miRNAs) as well as molecules exposed on EV external surfaces.

We have recently shown that exposure to Particulate Matter ≤ 10 µm (PM_10_) experienced during the 24 h preceding the blood drawing, is associated with an increased release of EVs and with elevated fibrinogen levels, in a large population of overweight subjects^12^. In addition, we investigated the relationship between EV release and PM exposure in 51 healthy volunteers, finding that PM-induced EV alterations in overweight subjects were more pronounced, with visible effect in all EV subtypes and particularly endothelial EVs^13^. Obesity is a strong risk factor for cardiovascular disease (CVD) as it modifies the effects of PM exposure on inflammation, oxidative stress, and acute phase response^14–17^.

In the present study, we investigated the effects of plasmatic EVs, isolated from healthy donors, on endothelial primary cell cultures. The approach of this study combines the natural complexity of human plasma EVs with a simple in vitro model, allowing us to evaluate the effect exerted by PM-induced EVs on the endothelial compartment, and the possible contribution of Body Mass Index (BMI) in determining the biological response of the endothelium.

## Material and methods

### Study design and participants

We enrolled six healthy, non-smokers volunteers without any previous medical history. None of the subjects was under chronic drug treatment and practiced agonistic physical activity. All the volunteers lived in Milan Metropolitan Area and had therefore comparable levels of outdoor baseline air pollution exposure. All subjects gave their written informed consent. The study was approved by the ethics committee named “Comitato Etico—Milano Area 2” of the Fondazione IRCCS Ca’ Granda Ospedale Maggiore Policlinico, 20122 Milan, Italy (approval number 1425), in accordance with principles of the Helsinki Declaration.

**Figure 1:**
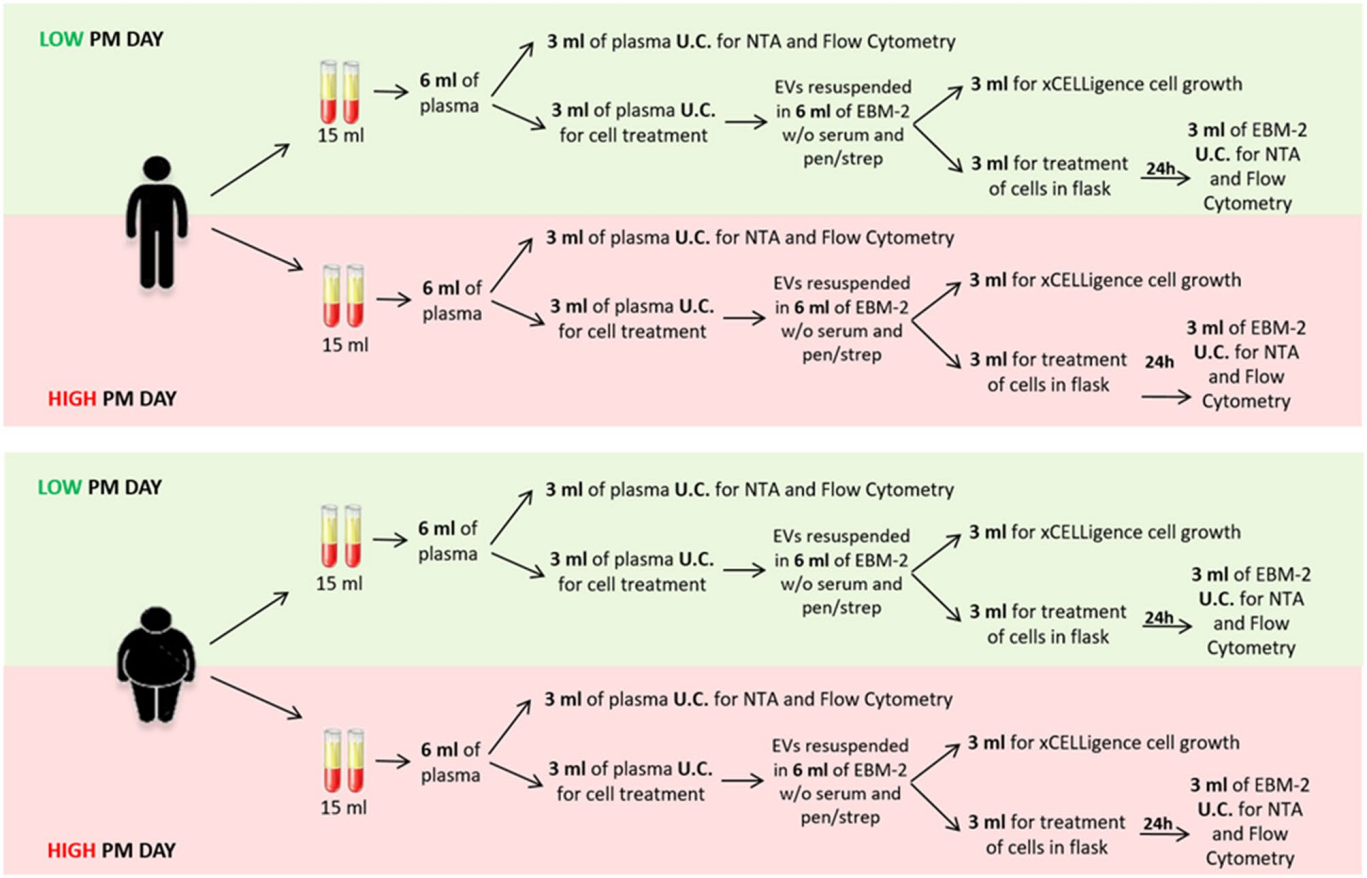
Experimental plan. *U.C.* ultra-centrifiuged; *NTA* nanoparticle tracking analysis; *EBM-2* endothelial basal medium 2; *EVs* extracellular vesicles.

For each subject, two blood drawings were performed in two different conditions: one the day after a high-PM_10_ day, and one the day after a low-PM_10_ day. Low-PM days were defined as days with PM levels below 50 µg/m^3^, whereas high-PM days had PM levels above 50 µg/m^3^. The time lag between high/low-PM_10_ days and blood drawing was chosen according to previous findings^12^. Each blood drawing was performed in fasting subjects at 9 a.m. in order to avoid circadian variations and processed within 2 h. A diagram describing the study design is reported in Fig. 1.

### PM exposure assessment

Daily information on the air quality (i.e. PM_10_) were estimated by Agenzia Regionale per la Protezione dell’Ambiente (ARPA) Lombardia (i.e. Regional Agency for Environment Protection), which collects data at a regional scale using the FARM (Flexible Air quality Regional Model) chemical-physical model of air quality^18^. This model is a three-dimensional Eulerian model that simulates the dispersion and chemical reactions of atmospheric pollutants. The system for forecasting pollutant concentrations is composed of a meteorological model powered by simulation data. In comparison, for the initial and boundary conditions, the outputs of the “Quale Aria” system are used^19^. Emissions are retrieved from regional, national, and European inventories. The domain of the simulation with the air quality model FARM covers the entire Lombardy region with a grid of 1 × 1 cm^2^ cells generated by the website, with municipality resolution to proper attribute to each subject the PM exposure experienced at home address the day before blood drawing. Finally, concentration data measured from the stations of the ARPA air quality network is integrated in the simulation results using interpolation techniques^20^. All participants were assigned pollutant levels that were estimated in the place of residence and in the Municipality of Milan.

### EVs analysis

Isolation, purification, and characterization of EVs were performed by following MISEV 2018 guidelines^21^. Detailed procedures and approaches are described in the Supplementary File.

### Endothelial cells isolation, culture and treatment

Endothelial primary cells were isolated from human cord blood^22,23^ and were established using Endothelial Basal Medium-2 (EBM-2) (Lonza, Inc.; Basel, Switzerland), enriched with 10% of Fetal Bovine Serum (FBS) (Sigma Aldrich, Inc.; Saint Louis, MO, USA) and 1% pen/strep (10 mg/mL) (Sigma Aldrich, Inc.; Saint Louis, MO, USA) and maintained in humidified incubators at 37 °C and 5% CO_2_. Culture conditions were set up and monitored through each experiment using the xCELLigence RTCA Single Plate System (Acea Bioscience, Inc.; San Diego, CA, USA). A day prior to stimulation with donor’s plasmatic EVs, cells were plated on gelatin 25 cm^2^ coated cell culture flasks (800,000 cells/flask). For each experiment, we treated three flasks of cells: each flask was treated with EVs isolated from subject’s plasma sample and re-suspended in 3 mL of EBM-2 without serum and antibiotics (Fig. 1). Cells treated only with EBM-2 without EVs were used as control. For each experiment, plasmatic EVs amount was normalized by volume, according to the MISEV2018 guidelines^21^. After 24 h of treatment, the cells were harvested and the medium was collected. After harvesting, cell viability assessment was performed by Propidium Iodide staining and flow-cytometry analysis (Miltenyi Biotec, Bergisch Gladbach, Germany).

### Isolation and purification of EVs

Whole blood was centrifuged at 1200×*g* for 15 min at room temperature to obtain platelet-free plasma. For isolation of plasma EVs, two aliquots of 3 mL of plasma for each subject were subsequently centrifuged at 1000, 2000, and 3000×*g* for 15 min at 4 °C. The obtained pellets were discarded to remove cell debris. EVs were then isolated from supernatants by ultracentrifugation at 110,000×*g* for 94 min at 4 °C in polypropylene ultracentrifuge tubes (Beckman Coulter; Brea, CA, USA) filled with PBS previously filtered through a 0.10-μm pore-size polyethersulfone filter (StericupRVP, Merck Millipore; Burlington, MA, USA). To carry out nanoparticles tracking analysis (NTA) and flow cytometry, the EV-rich pellet was resuspended in 0.5 mL of triple-filtered PBS (pore size 0.1 µm); the pellet for cell treatment was resuspended in 6 mL of EBM-2 (Lonza, Inc.) serum and penicillin–streptomycin free. For the isolation of EVs shed by endothelial cell cultures after treatments with plasmatic EVs, 3 mL of medium were collected from each flask, and subsequently centrifuged at 1000, 2000, and 3000×*g* for 15 min at 4 °C. EVs were isolated from supernatants by ultracentrifugation at 110,000×*g* for 9 h at 4 °C in polypropylene ultracentrifuge tubes (Beckman Coulter) and resuspended in 400 μL of triple-filtered PBS (pore size 0.1 µm). The methods here illustrated are described also in Pergoli et al.^12^ with major modifications, and are further detailed in the Supplementary File.

### Nanoparticle tracking analysis (NTA) of EVs

Numbers and dimensions of EVs were assessed by NTA, using the NanoSight NS300 system (Malvern Panalytical Ltd, Malvern, UK) as previously described^12^, which measures the Brownian motion of particles suspended in fluid and displays them in real time through a high sensitivity CCD camera. Five 30-s recordings were made for each sample. Collected data were analyzed with NTA software (Malvern Panalytical Ltd.), which provided high-resolution particle-size distribution profiles as well as measurements of the EV concentration.

### Flow cytometry on EVs

Flow cytometry on EVs Methods here detailed, are described also in Pergoli et al.^12^ with major modifications. EVs were characterized by High Resolution Flow Cytometry (MACSQuant, Miltenyi Biotec, Germany) according to the protocol for EV characterizations we previously developed and that is detailed in Supplementary File and at https://goo.gl/8un69P.

Briefly, samples’ acquisition was performed at the minimum speed flow (25 µL/min) using a MACSQuant Analyzer (Miltenyi Biotec). Sheath fluid was filtered through 0.1 μm pore size filter to further improve the signal-to-noise ratio. The fluorescent beads Fluoresbrite YG Carboxylate Microspheres Size Range Kit I (0.1, 0.2, 0.5, 0.75, and 1 μm) (Polysciences Inc, Warrington, Pennsylvania) were used to set the calibration gate in the FSC/FL1 and FSC/SSC dot plots. Using a side scatter (SSC) threshold of 10 arbitrary units, the lower sensitivity of the instrument was determined and the SSC and FITC voltages were set up. An overlap in the 100 nm beads population and the background noise was observed. In this way, it was possible to gate the MVs ≥ 200 nm diameter. 30 μL of sample was acquired on the MACSQuant Analyzer. Event numbers, analyzed at low flow rate and below 10.000 events/second, of equal sample volumes were counted. Information about concentration (No. events/μL) were calculated by the analyzer software. To verify the correctness of the count, we performed a serial dilutions measurement of different EV samples. r-value higher than 0.9 demonstrated the goodness of the experiment set up. From the data we set, the resuspending-volume of 500 µl of PBS resulted from the midpoint of the standard curve.

To analyze cell culture media-isolated EV integrity, for each experiment, an aliquot of resuspended EVs were stained with 0.2 μM 5(6)-carboxyfluorescein diacetate N-succinimidyl ester (CFSE) at 37 °C for 20 min in the dark. In order to assess the cellular origin of the EVs isolated from plasma, an immunophenotypization assay was performed for each sample using a panel of specific antibodies: CD14-APC (clone TÜK4), CD105-APC (clone 43A4E1), CD326 (EpCAM)-APC (clone HEA-125), CD61-APC (clone Y2/51), CD66abce-FITC (clone TET2). Furthermore, EVs isolated from cell culture media were incubated with CD61-APC (clone Y2/51), CD105-APC (clone 43A4E1) and CD62e-APC (clone REA280) antibodies. Each antibody aliquot was previously centrifuged at 17,000×*g* for 30 min at 4 °C to eliminate aggregates. A stained PBS control sample was used to detect the autofluorescence of each antibody. Quantitative multiparameter analysis of flow cytometry data was carried out by using FlowJo Software (Tree Star, Inc.; Ashland, OR, USA). Sample plots and gating strategies for each antibody are shown in the Supplementary File (Supplementary Figs. S1–S8).

### Statistical analysis

Descriptive statistics were performed on all variables. Categorical data are presented as frequencies and percentages. Continuous data were expressed as the mean ± SD or as the median and interquartile range (Q1-Q3), as appropriate. Normality assumption was verified by graphical inspection. Spaghetti and box plots were used to represent counts of EV subtypes by low and high PM exposure.

Poisson linear regression models for repeated measures were applied to evaluate the association between EV count in plasma subjects (total, CD61+, CD66+, CD14+, CD105+, EpCAM+) and PM group (High- vs Low-PM). We reported means with 95% CI and P-values. For each EV size, we estimated EV mean concentration and 95% CI in low and high PM group, with Poisson linear regression models for repeated measures. Due to the high number of comparisons, we used a multiple comparison method based on Benjamini–Hochberg False Discovery Rate (FDR) to calculate the FDR P-value. To display results of the analyses we used a series graph for EV mean concentrations of each PM group and vertical bar charts to represent FDR P-values and P-values. For the two graphs X axis was the size of EVs.

To evaluate whether the effect of PM exposure on EV characterization outcomes differs, depending the BMI values, subjects were stratified in two groups (BMI < or ≥ 25 kg/m^2^) and separate Poisson linear regression analysis for repeated measures were run. Interactions were tested by adding interaction term (BMI * PM) to the multivariable models. The same analysis was replicated for the number of EVs (total, CD62E+, CD105+ and their ratio) produced by cells treated with NW EVs or OW EVs (isolated both in condition of high and low PM exposure).

To evaluate the differences for each EV size in terms of EV average number produced by treated cells with NW EVs or OW EVs (isolated both at high and low PM exposure), we replicated the same graph and statistical models described above stratifying for BMI group. Statistical analyses were performed with SAS 9.4 software (SAS Institute Inc., Cary, NC).

### Ethics approval and consent to participate

All subjects gave their written informed consent, which had been approved by the ethics committee of the institution (approval number 1425), in accordance with principles of the Helsinki Declaration.

## Results

### Healthy donors’ characteristics and individual PM_10_ and PM_2.5_ exposure levels

The healthy donors we recruited (three males and three females) had a mean age (± SD) of 43 ± 11 years. According to Centers for disease control and prevention (CDC) definition, three subjects were classified as OW (mean BMI = 27.3 kg/m^2^ ± 1.7 kg/m^2^), and three were classified as NW (mean BMI = 23.0 kg/m^2^ ± 1.4 kg/m^2^). Descriptive statistics of PM_10_ and PM_2.5_ exposure levels from one day to seven days before blood drawings are reported in Table 1.

**Table 1:** Descriptive statistics of PM_10_ and PM_2.5_ exposure levels from day-1 to day-7 prior to blood drawings.

		Day-1	Day-2	Day-3	Day -4	Day-5	Day-6	Day-7
**PM**_**10**_** (µg/m**^**3**^**)**								
High PM	CTM municipality of domicile	100 (73—146)	112 ± 23	90 ± 16	83 ± 11	77 ± 9	69 ± 18	67 ± 23
	CTM municipality of Milan	119 ± 53	107 ± 26	79 ± 25	78 ± 5	75 ± 5	68 ± 6	89 ± 7
Low PM	CTM municipality of domicile	19 (18—24)	16 (11—21)	14 ± 4	18 ± 8	34 ± 22	30 ± 14	37 ± 24
	CTM municipality of Milan	34 ± 18	27 ± 19	17 ± 1	27 ± 4	38 ± 27	30 ± 24	40 ± 39
**PM**_**2.5**_** (µg/m**^**3**^**)**								
High PM	CTM municipality of domicile	88 ± 30	86 (82—104)	72 ± 11	69 ± 10	60 ± 6	50 ± 14	47 ± 12
	CTM municipality of Milan	98 ± 36	90 ± 22	72 ± 11	67 ± 12	64 ± 14	60 ± 16	67 ± 1
Low PM	CTM municipality of domicile	16 ± 9	15 ± 12	10 (9—14)	19 ± 6	29 ± 18	20 ± 13	28 ± 19
	CTM municipality of Milan	23 ± 13	23 (14—31)	15 ± 2	24 ± 8	30 (22—37)	21 ± 13	33 (23—44)

Continuous variables were expressed as mean ± standard deviation (SD) or as median [first quartile-third quartile], if not normally distributed.

### EV quantification in plasma of volunteers in low-PM and high-PM days

**Figure 2:**
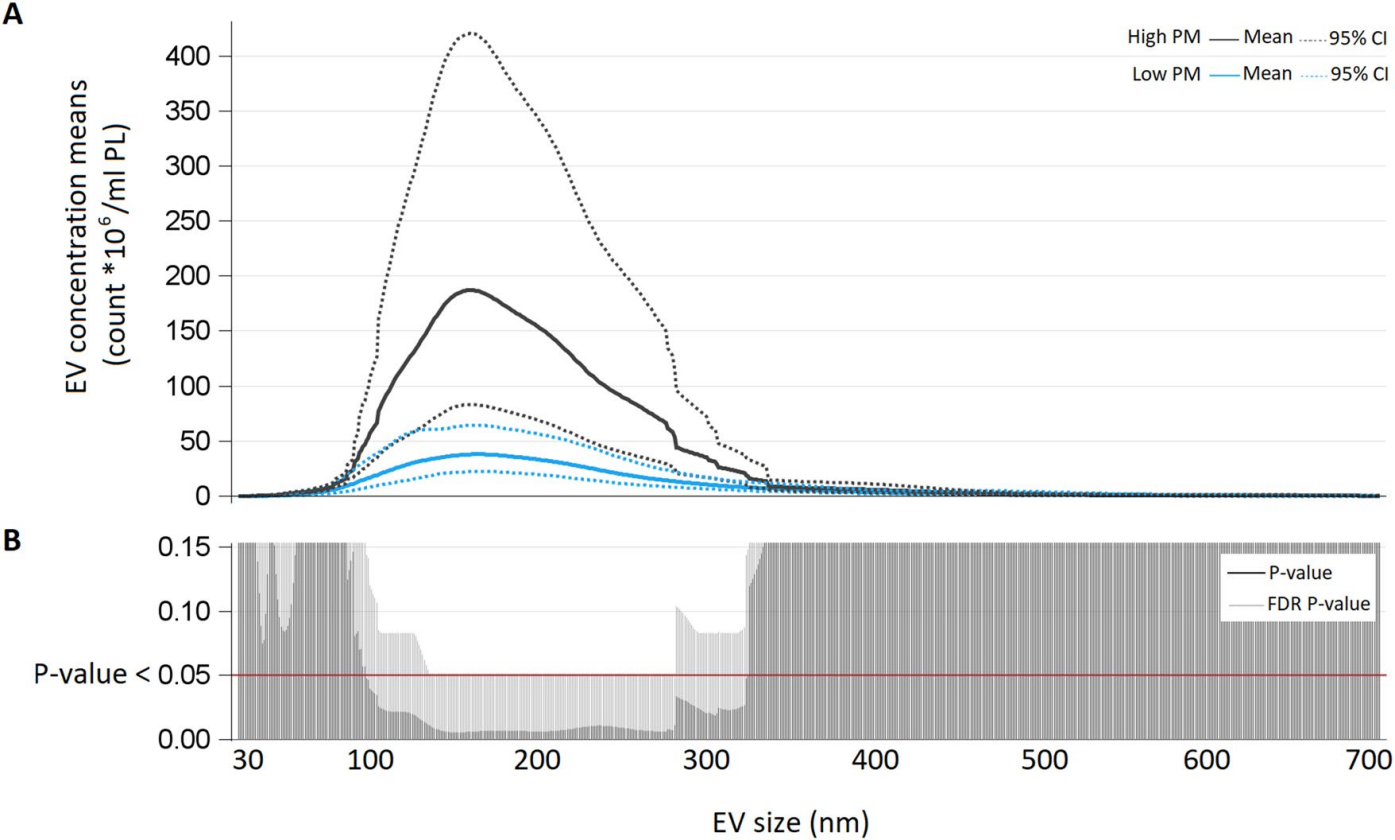
(**A**) EV concentration means (count *10^6^/mL PL) for each size (nm) from subjects exposed to low-PM and high-PM levels. (**B**) For each size P-value and False Discovery Rate. P-value from poisson regression models with repeated meausured are reported.

We measured the concentration of plasmatic EVs by NTA, and compared the sample obtained the day after a low-PM_10_ day with that obtained the day after a high-PM_10_ day. The day after a low-PM_10_ day, the mean of total EV concentration was 67*10^8^/mL plasma (PL) (95% CI 40*10^8^; 112*10^8^), whereas in the day after a high-PM_10_ day the mean was 264*10^8^/mL PL (95% CI 126*10^8^; 555*10^8^), P-value 0.0110. In order to better evaluate the possible EV concentration changes occurring after different PM_10_ levels of exposure, we considered the mean distribution of EV concentrations for each size (Fig. 2). In the upper part of the figure (Panel A), we reported for each EV size (from 30 to 700 nm) the mean concentration calculated in each condition (high or low PM_10_). Samples obtained at the day after high-PM_10_ days were characterized by a higher concentration of EVs of any size, in comparison to the samples obtained the day after a low-PM_10_ days. The lower part of the plot in Fig. 2B reports the P-values and FDR P-values obtained comparing each EV size concentration in the two conditions (i.e. high- and low-PM exposures) with Poisson linear regression models for repeated measures (n = 5) at each size.

### EV characterization in plasma of volunteers in low-PM and high-PM days

**Figure 3:**
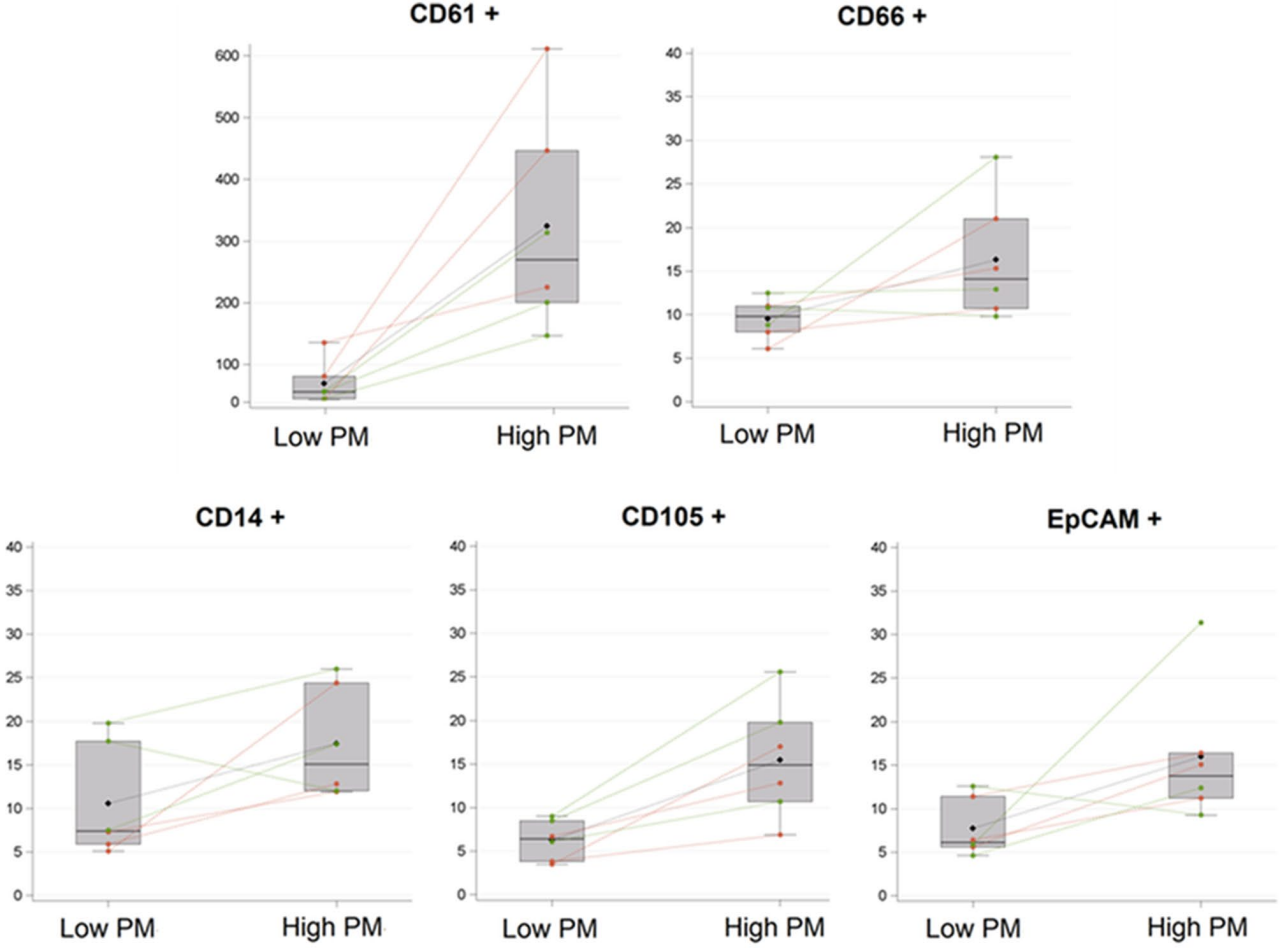
Spaghetti plot and box plot of EV subtypes from subjects exposed to low-PM and high-PM levels_._ EV subtypes are expressed as count *10^3^/mL PL. Red dots and lines represent OW subjects, green dots and lines represent NW subjects. Black dots and lines represent the mean of all subjects.

The panel of EV markers here evaluated was chosen according to our previous study^12^, where we demonstrated that they are specific for EV-releasing cells and possibly related to PM effects (Table 2). Six EV types were characterized: CD61 + EVs (released from platelets), CD66 + EVs (released from neutrophils), EpCAM + EVs (released from epithelial cells), CD105 + EVs (released from endothelium), CD14 + EVs (released from monocytes). A combination of spaghetti plot (reporting individual data) and box plot (reporting descriptive statistics) in the two groups, is reported in Fig. 3. Table 2 reports the means for each EV subtype, in the days at low and high PM exposure respectively. All the EV subtype concentrations were significantly higher the day after a high-PM day collected sample. The major difference was found for CD61 + EVs, which showed almost a fivefold increase the day after high-PM days (P-value < 0.0001).

**Table 2:** Mean concentrations of plasmatic EV subtypes deriving from subjects exposed to low-PM and high-PM levels.

EV characterization (*10^3^/mL PL)	Low-PM		High-PM		P-value
	Mean	95% CI	Mean	95% CI	
CD61 + (platelets)	66	(47;92)	309	(230;414)	< 0.0001
CD66 + (neutrophils)	10	(8;11)	16	(12;22)	0.0134
CD14 + (macrophages/monocytes)	10	(8;14)	17	(13;23)	0.0371
CD105 + (endothelium)	6	(5;7)	15	(12;20)	< 0.0001
EpCAM + (epithelium)	8	(6;11)	16	(11;23)	0.0100

Repeated measure analysis with poisson regression models (N = 6).

To better understand the role of BMI, which is reported to have an important biological role in modulating the effects of air pollution on EV release, we stratified our data in two groups: NW versus OW subjects. Interestingly, the differences observed between the day with low- and high-PM_10_ were confirmed also for the OW group, showing a significant increased production of all the considered EV types during the day with high PM_10_ level. On the contrary, NW subjects showed a significant increased production only for CD61+ and CD105 + EVs (P-value < 0.0001) (Table 3).

**Table 3:** Mean concentrations of plasmatic EV subtypes from subjects exposed to low-PM and high-PM levels, stratified for BMI (< or ≥ 25 kg/m^2^).

EV characterization (*10^3^/mL PL)	Normal weight: BMI < 25 kg/m^2^					Overweight: BMI ≥ 25 kg/m^2^					P-value for interaction
	(N = 3)					(N = 3)					
	Low-PM		High-PM		P-value	Low-PM		High-PM		P-value	
	Mean	95% CI	Mean	95% CI		Mean	95% CI	Mean	95% CI		
CD61 + (platelets)	52	(46;59)	220	(154;315)	< 0.0001	87	(53;142)	428	(281;650)	0.0002	0.7268
CD66 + (neutrophils)	11	(9;13)	17	(10;29)	0.1792	8	(6;11)	16	(12;21)	0.0121	0.6912
CD14 + (macrophages/monocytes)	15	(10;22)	18	(13;26)	0.3701	6	(5;7)	16	(11;24)	0.0003	0.0306
CD105 + (endothelium)	8	(7;9)	19	(13;27)	< 0.0001	5	(3;7)	12	(8;18)	0.0003	0.7287
EpCAM + (epithelium)	8	(5;13)	18	(9;33)	0.0967	8	(5;11)	14	(12;17)	0.0001	0.6597

Repeated measure analysis with poisson regression models.

### Endothelial EVs production after treatments

We treated endothelial cells by using the EVs isolated from the same volume of plasma samples and previously characterized. In order to take into account the expected response variability due to BMI contribution, we considered separately cells stimulated with NW group EVs and OW group EVs. In all the experiments, the percent of dead cells was between 5.7 and 1.7%. Following vitality assay, we did not observe any significant difference between both OW- and NW- plasmatic-EVs-treated cells and controls (untreated cells) at both high and low PM levels (Supplementary Fig. S9).

**Figure 4:**
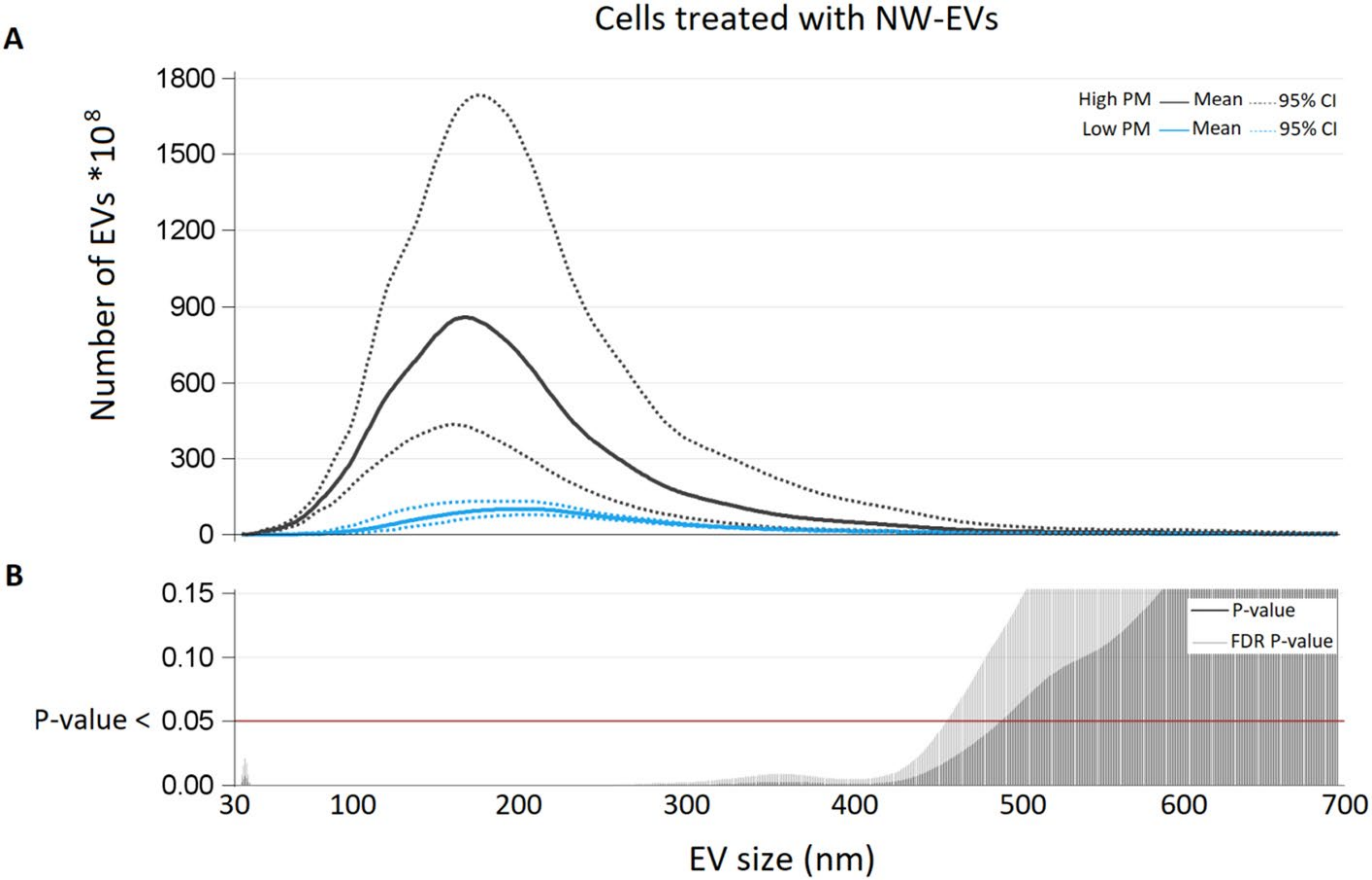
(**A**) Number of EVs (*10^8^) for each size (nm) from NW subjects exposed to low-PM and high-PM levels. (**B**) For each size P-value and False Discovery Rate P-value from poisson regression models with repeated meausured are reported.

**Figure 5:**
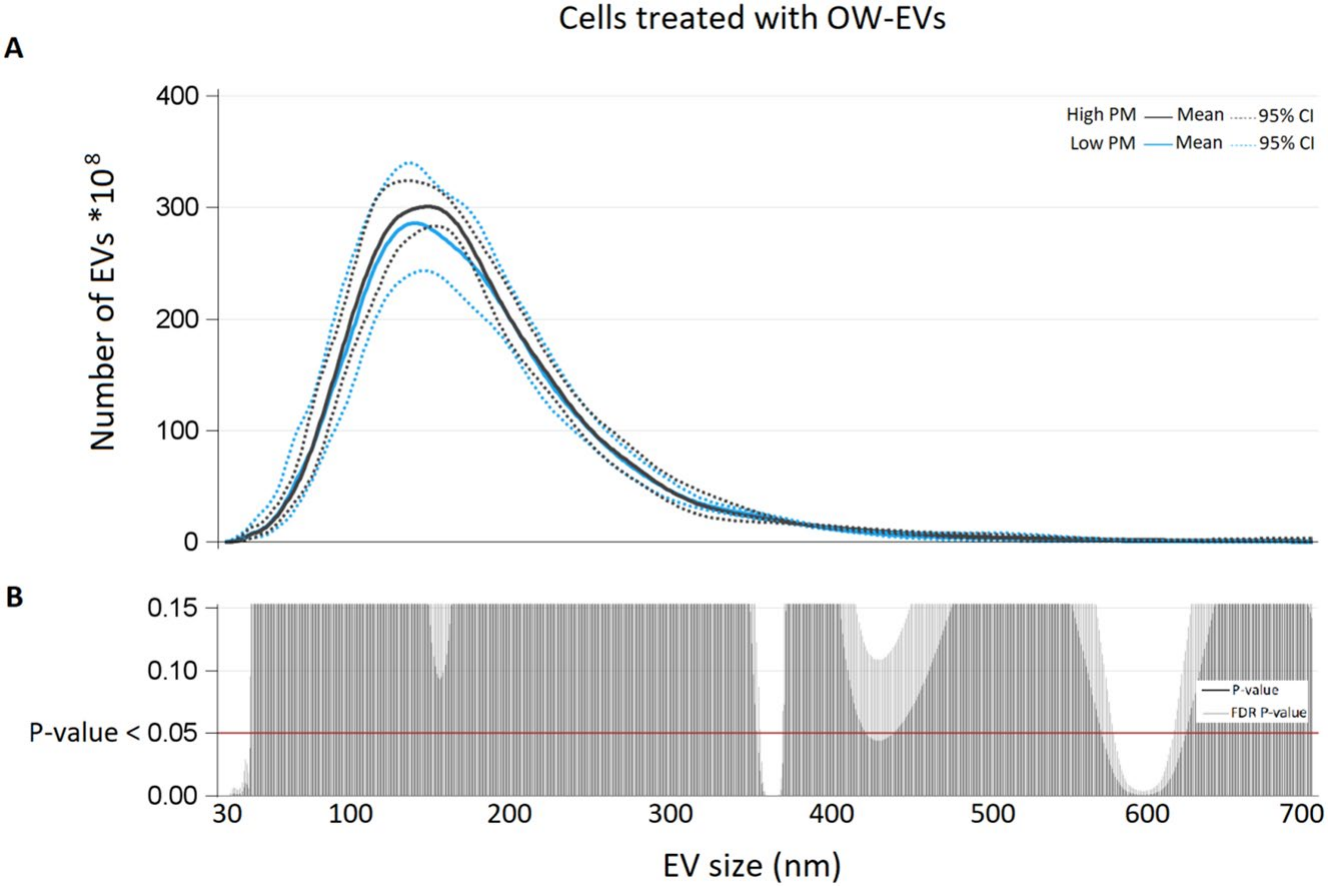
(**A**) Number of EVs (*10^8^) for each size (nm) produced by cells treated with EVs from OW subjects, exposed to low-PM and high-PM levels. (**B**) For each size P-value and False Discovery Rate P-value from Poisson regression models with repeated measured are reported.

We observed a tenfold increased endothelial EV release between sample treated with NW-high-PM EVs compared to those treated with NW-low-PM EVs (p-value 0.0110) (Fig. 4). The mean of total EVs released after treatment with OW-low-PM EVs was 434*10^10^ (95% CI 382*10^10^; 493*10^10^), while the mean of the ones total OW-high-PM EVs was 453*10^10^ (95% CI 423*10^10^; 485*10^10^), and the difference was not statistically significant (P-value 0.1551) (Fig. 5). Interaction between PM exposure and BMI was formally performed to assess BMI modification effects and resulted to be statistically significant (P-value < 0.0001).

**Figure 6:**
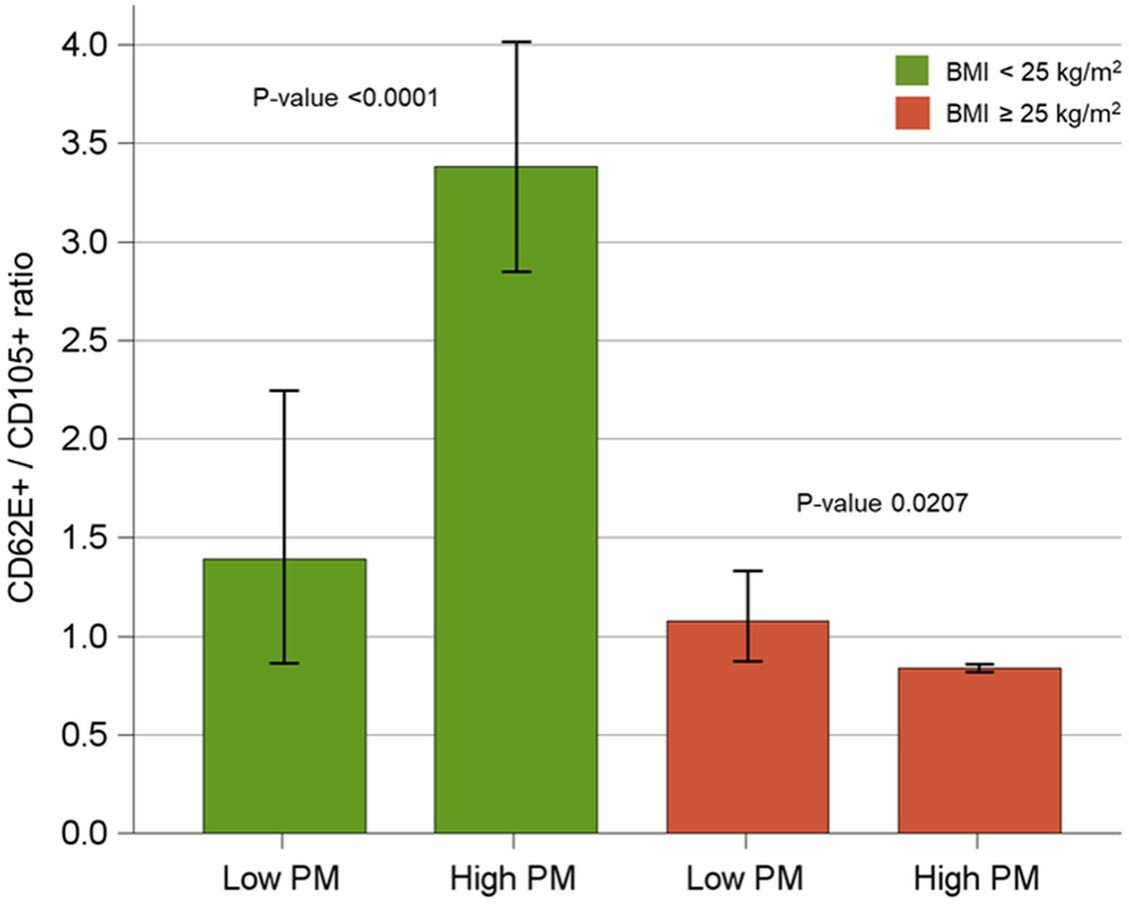
Average changes of CD62e+/CD105 + ratio with 95% CI after cells treatments with low- and high-PM plasmatic EVs stratified for BMI (< or ≥ 25 kg/m^2^). Repeated measure analysis with Poisson regression model.

To estimate the amount of plasmatic EVs in the collected cell cultures media, CD61 + EVs were evaluated, as they were the most representative plasmatic EV subtype previously observed in plasma samples (Table 2), and no CD61 + EVs were detected. This evidence suggests that all the EVs were incorporated into culture cells. In order to evaluate endothelial response, we quantified CD62e + EVs and CD105 + (Table 4), which are biomarkers for the whole and the activated endothelium respectively. CD62e+/CD105 + EVs ratio was also calculated, to estimate endothelial activation (Table 4). Amounts of cellular EVs are expressed as fold change between EVs from plasmatic-EVs-treated cells and controls (untreated cells). Cells treated with NW-EVs showed an increased endothelial activation in the high-PM day, whereas cells treated with OW-EVs showed a reduced endothelial activation in the day characterized by an high-PM day (Fig. 6).

**Table 4:** Mean of cellular EV subtypes isolated after treatments with EVs from subjects exposed to low-PM and high-PM levels, stratified for BMI (< or ≥ 25 kg/m^2^).

EV characterization (*10^3^/mL PL)	Cells treated with NW EVs: BMI < 25 kg/m^2^					Cells treated with OW EVs: BMI ≥ 25 kg/m^2^					P-value for interaction
	(N = 3)					(N = 3)					
	Low-PM		High-PM		P-value	Low-PM		High-PM		P-value	
	Mean	95% CI	Mean	95% CI		Mean	95% CI	Mean	95% CI		
Number of CD105 + EV *10^5^ (endothelium)	197	(147;264)	126	(118;135)	0.0004	94	(79;112)	200	(188;218)	< 0.0001	< 0.0001
Number of CD62E + EV *10^5^ (activated endothelium)	245	(191;313)	425	(363;497)	0.0007	99	(97;100)	169	(159;179)	< 0.0001	0.9383
Ratio CD62E+/CD105+	1.39	(0.86;2.25)	3.38	(2.85;4.02)	< 0.0001	1.08	(0.87;2.25)	0.84	(0.82;0.86)	0.0207	< 0.0001

Repeated measure analysis with poisson regression models. Amounts of cellular EVs are expressed as fold change between EVs isolated from culture media of treated and untreated cells (taken as controls).

## Discussion

Short-term PM exposure is associated with an increased release of EVs^12,13,24^, which are considered to be important mediators of the cross-talk between cells^10^. A growing body of evidence shows that the effects of PM exposure is associated with endothelial dysfunction, suggesting that EVs may be important players in this mechanism^12,13^.

In the present study, we investigated the impact of plasmatic EVs isolated from healthy donors on endothelial primary cells. We considered PM levels of the days prior to blood drowings as we and others previously demonstrated positive short-term PM_10_ exposure (day -1) effects on EVs^12,13,24^. We confirmed that the concentration of plasma EVs was significantly increased the day after high-PM_10_ days compared to that in the day after low-PM_10_ days, as already reported. Our results support the hypothesis that EV release may represent a molecular mechanism that mediates short-term systemic response to PM exposures as well as to other environmental stimuli.

In order to identify the cellular origin of plasmatic EVs in the analyzed samples, they were further characterized by flow cytometry, taking advantage of a panel of markers we previously demonstrated being EV-releasing cells related to PM effects^12^. Interestingly, the concentration of all the investigated plasmatic EV types was significantly higher in the high-PM_10_ days, and the most significant difference was observed for CD61 + EVs. CD61 is a marker for platelets^25,26^ and interactions of platelet-derived EVs with leukocytes are mostly reported in the context of inflammation^27^. Moreover, platelet-derived EVs seem to diverge in glycoprotein expression levels and also differ in modulating monocyte activities, depending on the ageing and/or activation status of the megakariocytes they derive from^28^. However, how platelet-derived EVs contribute to platelet communication with their surroundings is still unclear^29^.

Since BMI has an important biological role in modulating the effects of air pollution on EVs release^12,13,30^, we stratified the enrolled subjects into two groups (NW and OW). NW subjects showed a statistically significant increased production only of CD61 + and CD105 + EVs, suggesting a preferential response of platelets and endothelium to PM exposure in physiological condition. As it has already widely documented that PM exposure plays a central role in mediating the development of CVDs^31–33^, we speculate that platelets and endothelium may display protective mechanisms toward environmental stimuli (i.e. PM) also by the increase of EVs release.

As inflammatory response caused by high PM exposure induces endothelial activation^34,35^, we investigated the impact of plasmatic EVs from each recruited sample at a high- and a low-PM day on primary endothelial cells. Following the calculation of CD62e+/CD105 + EVs ratios as a measure of endothelial activation, we observed that cells treated with NW-high-PM EVs showed an increased endothelial activation. On the contrary, cells treated with OW-high-PM EVs showed a reduced endothelial activation. It is well known that under physiological conditions the stimulation of the endothelium leads to cardiovascular protective effects by relaxing media-smooth muscle cells and preventing leukocyte adhesion and migration into the arterial wall, muscle cell proliferation, platelet adhesion and aggregation, and adhesion molecule expression^36^. In this light, plasmatic EVs released after PM exposure may communicate to endothelial cells, thus promoting the cross-talk between endothelium and the surrounding environment such as immune and muscle cells. Therefore, the increase of CD62e + EVs release by endothelial cells after treatment with NW EVs exposed to high-PM may be biologically relevant. On the othe hand, it is well known that high BMI causes chronic low-grade inflammation^37^. Thus, while NW subjects showed a low and targeted reactivity to PM exposure^12^, we speculate that the persistent low-grade inflammation in OW subjects may determine a sort of tolerance to EV biological effects. This condition may bring to a lack of endothelial activation, probably due to an unproper response to environmental stimuli, such as exposure to PM. However, this research field is at its infancy and further studies are necessary to unveil the mechanisms underlying the incapability of blood EVs produced by OW subjects exposed to high PM levels to properly activate endothelial cells. We did not evaluate the levels of adipocytes-derived-EVs although they are known to be involved in the cross-talk between adipose tissue and endothelium^38^. However, as they are released in plasma^39^, they may contribute to determine different endothelial activation in NW- compared to OW-subjects.

Although this study involved six subjects, the consistency of our results is supported by the data obtained in the two different stratified groups. Moreover, the integrated approach used in this study allowed to model in vitro the effects mediated by the in vivo physiological response in terms of EV release after PM exposure.

## Conclusion

Our findings contribute to explain how PM exposure is associated with an increased risk of CVDs in OW subjects by elucidating one of the mechanisms involved in endothelial activation after PM exposure.

## Supplementary information

Supplementary Information.

## Abbreviations

EVs: Extracellular Vesicles
PM: Particulate Matter
PM_10_: Particulate Matter ≤ 10 µm
PM_2.5_: Particulate Matter ≤ 2.5 µm
CVD: CardioVascular Disease
NW: Normal Weight subjects
OW: Over Weight subjects
BMI: Body Mass Index
CV: CardioVascular
miRNAs: MicroRNAs
ARPA: Agenzia Regionale per la Protezione dell’Ambiente Lombardia
CTM: Chemical Transport Model
EBM-2: Endothelial Basal Medium-2
NTA: Nanoparticle tracking
CDC: Centers for Disease Control and prevention
CFSE: 5(6)-Carboxyfluorescein diacetate *N*-succinimidyl ester
FDR: False Discovery Rate
PL: Plasma
