# Supplementary material for: Blood-derived extracellular vesicles isolated from healthy donors exposed to air pollution modulate in vitro endothelial cells behavior
Source: Sci Rep. 2020 Nov 18;10:20138. doi: 10.1038/s41598-020-77097-9 (PMC7674466; doi:10.1038/s41598-020-77097-9)
Supplement: Supplementary file 1 — Supplementary Information. [file 41598_2020_77097_MOESM1_ESM.docx]

**BLOOD-DERIVED EXTRACELLULAR VESICLES ISOLATED FROM HEALTHY DONORS EXPOSED TO AIR POLLUTION MODULATE *in vitro* ENDOTHELIAL CELLS BEHAVIOR**

Federica Rota^1^**^#^**, Luca Ferrari^1^**^#^**, Mirjam Hoxha^1^, Chiara Favero^1^, Rita Antonioli^1^, Laura Pergoli^1^, Maria Francesca Greco^2^, Jacopo Mariani^1^, Lorenza Lazzari^3^, Valentina Bollati^1^**^*^**

^1^ EPIGET LAB, Department of Clinical Sciences and Community Health, Università degli Studi di Milano, Milan, Italy.

^2^ Department of Pharmacological and Biomolecular Sciences, Università degli Studi di Milano, Milan, Italy.

^3^ Cell Factory, Laboratory of Regenerative Medicine, Department of Services & Preventive Medicine, Fondazione IRCCS Ca' Granda Ospedale Maggiore Policlinico, Milano, Italy.

^#^The authors equally contributed to this paper

***Correspondence to**

Valentina Bollati, PhD

EPIGET LAB, Department of Clinical Sciences and Community Health

Università degli Studi di Milano

Via San Barnaba, 8

20122 Milan, Italy

e-mail: [valentina.bollati@unimi.it](mailto:valentina.bollati@unimi.it)

phone: +39 02 503 20147

**Supplementary materials and methods**

**MISEV 2018 guidelines compliance**

|  | **Section title** | **Required information according to MISEV2018** | **Mandatory requirement** | **Not applicable/not available** | **Our approach** | **Compliance with MISEV2018 requirements** |
| --- | --- | --- | --- | --- | --- | --- |
| 1 | Nomenclature | The term extracellular vesicle (EV) can be used with demonstration of extracellular (no intact cells) and vesicular nature per these characterization and function | YES |  | As explained in section 4 and 5, the term extracellular vesicle (EV) has been used in the manuscript | YES |
| 2a | Collection and pre-processing (tissue culture conditioned medium) | General cell characterization | YES |  | Human endothelial primary cells were isolated from cord blood (Eggerman et al., 2003 PMID: 12757882; Huizer at al., 2017 PMID: 28910385 ) | YES |
| 2a | Collection and pre-processing (tissue culture conditioned medium) | Medium used before and during collection (additives, serum, other) | YES |  | - Human endothelial primary cells were grown and sub-cultured in Endothelial Basal Medium-2 (EBM-2) (Lonza, Inc. >Basel, Switzerland), containing 10% v/v fetal bovine serum and antibiotics (50 U/mL penicillin; 50µg/mL streptomycin; Sigma Aldrich, Inc.; Saint Louis, MO, USA). | YES |
| 2a | Collection and pre-processing (tissue culture conditioned medium) | Exact protocol for depletion of EVs from additives in collection medium | YES |  | EVs were obtained after 24 hours cell culture in culture medium w/o serum and pen/strep conditioned by cells | YES |
| 2a | Collection and pre-processing (tissue culture conditioned medium) | Nature and size of culture vessels, and volume of medium during conditioning  e) specific culture conditions (treatment, % O2, coating,polarization…) before and during collection | YES |  | A day prior to stimulation with donor’s plasmatic EVs, cells were plated on gelatin 25 cm^2^ coated cell culture flasks (800,000 cells/flask). | YES |
| 2a | Collection and pre-processing (tissue culture conditioned medium) | Number of cells/ml and % of live/ dead cells at time of collection | YES |  | 800,000 cells were seeded and the percentage of dead cells at time of collection was below 10%. | YES |
| 2a | Collection and pre-processing (tissue culture conditioned medium) | Frequency and interval of Conditioned Medium harvest | YES |  | EVs were obtained after 24 hours cell culture in culture medium w/o serum and pen/strep conditioned by cells | YES |
| 2b and 2c | Collection and pre-processing (Biofluids or tissues) | Donor status if available (age, sex, food/water  intake, collection time, disease, medication, other) | YES |  | Each blood drawing was performed in healthy, non-smokers and fasting volunteers without any previous medical history, at 9 a.m., and the blood was processed within 2 hrs | YES |
| 2b and 2c | Collection and pre-processing (Biofluids or tissues) | Volume of biofluid or volume/mass of tissue sample  collected per donor | YES |  | For each subject, two blood samples were collected in two EDTA tubes (7 mL each) | YES |
| 2b and 2c | Collection and pre-processing (Biofluids or tissues) | Total volume/mass used for EV isolation (if pooled  from several donors | YES |  | EVs isolation was performed starting from two aliquots of 3 mL of plasma for each subject. One of the two EVs pellet obtained from each subject were resuspended in 0.5 mL of PBS triple filtered (pore size 0.1 µm). The other one was resuspended in 6 mL of EBM-2 w/o serum and pen/strep and used for cell treatment. | YES |
| 2b and 2c | Collection and pre-processing (Biofluids or tissues) | All known collection conditions, including additives,  at time of collection |  |  | Blood samples were collected in 7 mL EDTA tubes | YES |
| 2b and 2c | Collection and pre-processing (Biofluids or tissues) | Pre-treatment to separate major fluid-specific contaminants  before EV isolation |  |  | Blood was centrifuged at 1200 × *g* for 15 min at room temperature to obtain platelet-free plasma. After that, two aliquots of 3 mL of plasma for each subject were subsequently centrifuged at 1000, 2000, and 3000 × *g* for 15 min at 4 °C. The obtained pellets were discarded to remove cell debris. | YES |
| 2b and 2c | Collection and pre-processing (Biofluids or tissues) | Temperature and time of biofluid/tissue handling  before and during pre-treatment |  |  | Each sample was processed within 2 hrs and maintained at 4 °C | YES |
| 2b and 2c | Collection and pre-processing (Biofluids or tissues) | For cultured tissue explants: volume, nature of  medium and time of culture before collecting conditioned  medium |  | N/A |  |  |
| 2b and 2c | Collection and pre-processing (Biofluids or tissues) | For direct tissue EV extraction: treatment of tissue  to release vesicles without disrupting cells |  | N/A |  |  |
| 2d | Storage and recovery | Storage and recovery (e.g., thawing) of CCM, biofluid, or tissue before EV isolation (storage temperature, vessel, time; method of thawing or other sample preparation) | YES |  | Culture medium were processed within 1 hour from harvesting and maintained at 4 °C. | YES |
| 2d | Storage and recovery | Storage and recovery of EVs after isolation (temperature, vessel, time, additive(s)…) | YES |  | Isolated EVs were immediately characterized by flow cytometry and Nanosight. Zebrafish injection was performed within 24 hours from medium harvesting. | YES |
| 3 | EV separation and concentration | Centrifugation: reference number of tube(s), rotor(s), adjusted k factor(s) of each centrifugation step (= time+  speed+ rotor, volume/density of centrifugation conditions), temperature, brake settings | YES |  | We applied a differential ultracentrifugation with previous lower-speed steps. 8 mL of medium were collected in Corning 15 mL PP Centrifuge Tubes, and centrifuged at 1000, 2000, and 3000 × g for 15 min at 4 °C (Haereus Labofuge 400R, Hanau, Germany). The obtained pellets were discarded to remove cells, apoptotic bodies and cell debris. EVs were then isolated from supernatants by ultracentrifugation at 110,000 × g for 9 hours at 4 °C in polypropylene ultracentrifuge tubes (Quick-Seal ultra-clear centrifuge tubes, Beckman Coulter; Brea, CA, USA) rotor MLA-55 (Beckman Coulter), filled with PBS previously filtered through a 0.10-μm pore-size polyethersulfone filter (StericupRVP, Merck Millipore; Burlington, MA, USA). Our method is included in the category “Intermediate recovery, intermediate specificity = mixed EVs with limited non-EV components” | YES |
| 3 | EV separation and concentration | Density gradient: nature of matrix, method of generating gradient, reference (and size) of tubes, centrifugation speed and time (with brake specified), method and volume of fraction recovery. |  | N/A |  |  |
| 3 | EV separation and concentration | Chromatography: matrix (nature, pore size,…), loaded sample volume, fraction volume, number |  | N/A |  |  |
| 3 | EV separation and concentration | Precipitation: reference of polymer, ratio vol/vol or weight/vol polymer/fluid, time/temperature of incubation, time/speed/temperature of centrifugation |  | N/A |  |  |
| 3 | EV separation and concentration | Filtration: reference of filter type (=nature of membrane, pore size…), time and speed of centrifugation, volume before/after (in case of concentration) |  | N/A |  |  |
| 3 | EV separation and concentration | Antibody-based : reference of antibodies, mass Ab/amount of EVs, nature of Ab carrier (bead, surface) and amount of Ab/carrier surface |  | N/A |  |  |
| 3 | EV separation and concentration | Other…: all necessary details to allow replication |  | N/A |  |  |
| 3 | EV separation and concentration | Additional step(s) to concentrate, if any |  | N/A |  |  |
| 3 | EV separation and concentration | Additional step(s) to wash matrix and/or sample, if any |  | N/A |  |  |
| 4a | EV characterization, Quantification | Volume of fluid, and/or cell number, and/or tissue mass used to isolate EVs | YES |  | 3 ml of culture medium for each flask were collected from each flask and EVs were isolated from supernatants by ultracentrifugation at 110,000 × g for 9 hrs at 4 °C in polypropylene ultracentrifuge tubes (Beckman Coulter) and pellet was resuspended in 400 μL of triple-filtered PBS. | YES |
| 4a | EV characterization, Quantification | Global quantification by at least 2 methods: protein amount, particle number, lipid amount, expressed per volume of initial fluid or number of producing cells/mass of tissue | YES |  | In order to quantify the total number of EVs we applied the two following approaches:  1) Nanoparticle tracking analysis by NanoSight NS300 system (Malvern Panalytical Ltd, Malvern, UK). Five 30-s recordings were made for each sample. Collected data were analyzed with NTA software (Malvern Panalytical Ltd.), which provided high-resolution particle-size distribution profiles as well as measurements of the EV concentration.  2) High resolution Flow cytometry by MACSQuant, Miltenyi Biotec. In order to analyze EV integrity, 60 µl aliquots were stained with 0.2 μM 5(6)-carboxyfluorescein diacetate N-succinimidyl ester (CFSE) at 37 °C for 20 min in the dark. CFSE is a  cell permeant, non-fluorescent pro-dye. If incorporated into intact EVs, which contain esterases as live cells, the acetate groups of CFSE is cleaved producing a membrane-impermeant molecule with green fluorescence. | YES |
| 4a | EV characterization, Quantification | Ratio of the 2 quantification figures | YES | N/A |  |  |
| 4b | EV characterization, General Characterization | At least three positive protein markers of EVs, including at least one transmembrane/lipid bound protein and one cytosolic protein  At least one negative protein marker | YES |  | The following antibodies have been used to measure positive protein markers of plasmatic EVs:  anti- CD14-APC (clone TÜK4) (Miltenyi Biotec), anti-CD105-APC (clone 43A4E1) (Miltenyi Biotec), anti-CD326 (EpCAM)-APC (clone HEA-125) (Miltenyi Biotec), anti-CD61-APC (clone Y2/51) (Miltenyi Biotec), anti-CD66abce-FITC (clone TET2), (Miltenyi Biotec);  Transmembrane, tissue specific and focus of the present study.  The following antibodies have been used to measure positive protein markers of cell cultures media EVs:  Anti-CD61-APC (clone Y2/51) (Miltenyi Biotec),  Transmembrane, tissue aspecific  Anti-CD105-APC (clone 43A4E1) (Miltenyi Biotec),  Anti-CD62e-APC (clone REA280);  Transmembrane, tissue specific and focus of the present study.  CFSE staining was used to assess cytosolic esterase activity. As negative control, representative EVs samples were incubated with Tween20 (P1379; Merk, Sigma-Aldrich) ON at 37 °C, and then incubated with CFSE as described above. Positivity for CFSE fluorescence was evaluated by High Resolution Flow cytometry analysis | YES |
| 4c | Single EV characterization | Images of single EVs by electron microscopy | YES |  | Transmission Electron Microscopy (TEM) analysis was performed on random samples as quality control. | YES |
| 4c | Single EV characterization | Non-image-based method analysing large numbers of single EVs: Non-image-based method analysing large numbers of single EVs: NTA, TRPS, FCS, high-resolution flow cytometry, multi-angle light-scattering, Raman spectroscopy, etc. | YES |  | NTA and , high-resolution flow cytometry were performed | YES |
| 5 | Functional studies | Dose-response assessment | YES |  | EVs were normalized by volume | YES |
| 5 | Functional studies | Negative control = nonconditioned medium, biofluid/tissue from control donors, as applicable | YES |  | Non-conditioned medium was used as negative control. | YES |
| 5 | Functional studies | Quantitative comparison of functional activity of total fluid, vs EV-depleted fluid, vs EVs (after high recovery/low specificity separation) | YES |  | Quantitative comparison was performed for each experiment, by establishing a negative control with only EBM-2 with no plasmatic EVs added. | YES |
| 5 | Functional studies | Quantitative comparison of functional activity of EVs vs other EPs/fractions after low recovery/high specificity separation | YES | N/A |  |  |
| 5 | Functional studies | Quantitative comparison of activity of EV subtypes (if subtype-specific function claimed) | YES |  | Functional effects were evaluated by calculating the ratio of CD62e+/ CD105+ EVs isolated from cell culture media. | YES |
| 5 | Functional studies | Extent of functional activity in the absence of contact between EV donor and EV recipient | YES |  | To estimate the amount of plasmatic EVs in the collected cell cultures media, CD61+ EVs were evaluated, as they were the most representative plasmatic EV subtype previously evaluated, and no CD61+ EVs were detected | YES |
| 6 | Reporting | Submission of data (proteomic, sequencing, other) to relevant public, curated databases or open-access repository | YES | N/A |  |  |

**Supplementary methods for High Resolution Flow Citometry**

Preparation of microvesicles from blood is detailed at

<https://www.miltenyibiotec.com/_Resources/Persistent/312f1e5e920ed7504093d47943f8561aa3a9079c/Characterization%20of%20microvesicles.pdf>

Microvesicles have been isolated from blood samples within two hours after blood drawing.

**Preparation of extracellular vesicles from primary endothelial cells:**

The protocol here described is similar to that previously reported by Ferrari et al., 2019 (Int J Mol Sci. 2019 Jul 26;20(15):3669. doi: 10.3390/ijms20153669) and adapted for the isolation of extracellular vesicles from primary endothelial cells.

1. Draw 6 mL of cell medium into 15 mL tubes.

3. Remove cell debris from medium by serial centrifugation at 1,000, 2,000, and 3,000 × g for 15 minutes at 4 °C.

4. Transfer 3 mL of supernatant into an ultracentrifuge tube and fill up with 0.10 µm pore size membrane-filtered PBS.

5. Ultracentrifuge sample at 110,000 × g for 9 hours at 4 °C.

6. Resuspend the ultracentrifuged pellet with 400 µL triple 0.10 µm pore size membrane-filtered PBS.

7. Transmission electron microscopy (TEM) was used to check the morphology of extracellular vesicles and aggregates.

8. 100 µL of unstained sample were used for NanoSight analysis to check for aggregates and the size and concentration of extracellular vesicles.

**Staining of sample**

9. 60 µL of sample and 60 µL of triple 0.10 µm pore size membrane-filtered PBS (control sample) were stained with 0.02 µM CFSE at 37 °C for 20 minutes in the dark.

10. The CFSE stained sample and the control sample were incubated with 6 µL of CD61, CD62e and CD105 antibodies in the dark for 20 minutes at 4 °C.

Note: Before use, the antibodies were centrifuged at 17,000 × g for 30 minutes at 4 °C to eliminate aggregates.

**Data acquisition and analyzes using the MACSQuant Analyzer**

Note: Sheath fluid was filtered using 0.1 μm pore size filter to further improve the signal-to-noise ratio.

12. Unstained triple 0.10 μm pore size membrane-filtered PBS was acquired to evaluate the buffer background noise.

13. The stained PBS control sample was acquired to detect the autofluorescence of the antibodies (Supplementary Figures S1-S8).

14. 30 µL of unstained sample were acquired to detect the sample auto-fluorescence (Supplementary Figures S1-S8).

15. The Fluoresbrite® Carboxylate Size Range Kit I (0.2, 0.5, 0.75, and 1 µm) was used to set the calibration gate in the FSC/FL1 and FSC/SSC dot plots on MACQSQuant Analyzer.

16. 30 μL of double stained sample were acquired on the MACSQuant Analyzer.

17. Quantitative multi-parameter analysis of flow cytometry data was carried out using FlowJo Software (Tree Star, Inc.).

**Supplementary Figures**

**Supplementary Figure S1:** EpCAM gating protocols for plasmatic EVs. A-C negative control with PBS; D-F Plasma samples. A, D): scatter plots; B, E): dot plots with gating for CFSE fluorescence; C, F): dot plots for experimental sample (scatter+ CFSE + ANTI-EpCAM antibody); D: dot plots for scatter+ ANTI-EpCAM antibody.

**
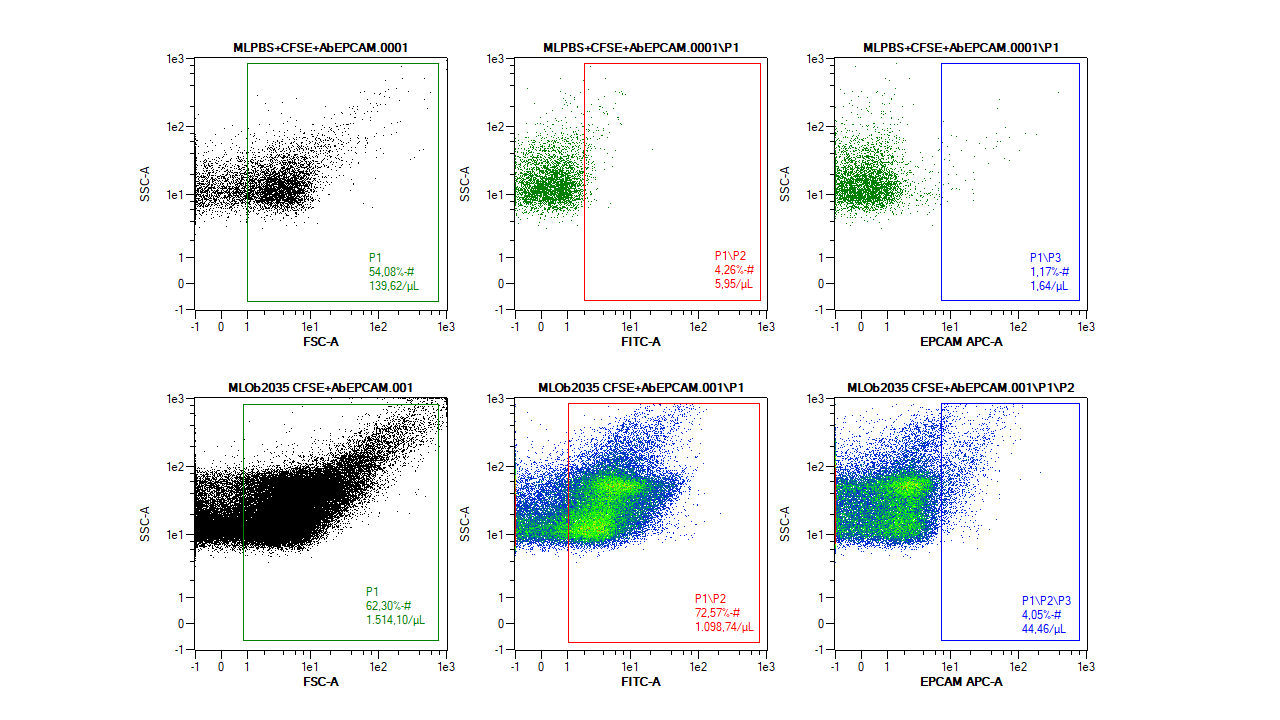
**

**Supplementary Figure S2:** CD66 gating protocols for plasmatic EVs. A-C negative control with PBS; D-F Plasma samples. A, D): scatter plots; B, E): dot plots with gating for CFSE fluorescence; C, F): dot plots for experimental sample (scatter+ CFSE + ANTI- CD66 antibody); D: dot plots for scatter+ ANTI- CD66 antibody.


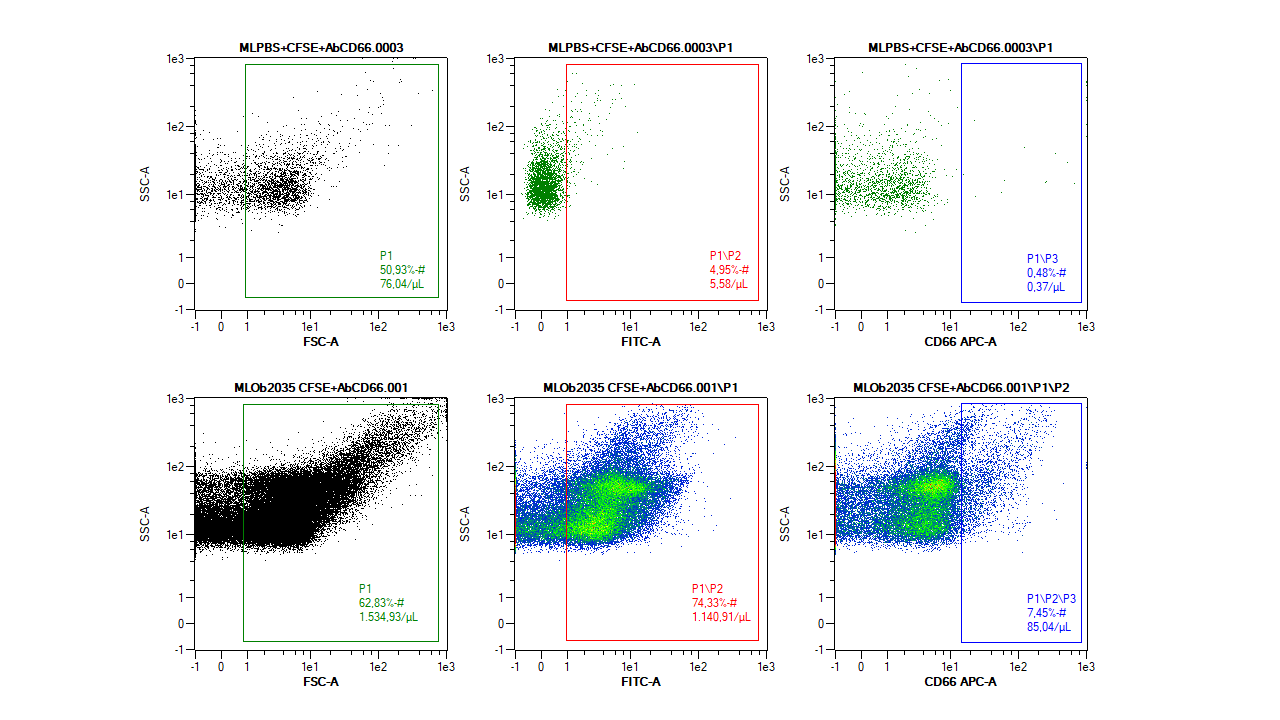


**Supplementary Figure S3:** CD14 gating protocols for plasmatic EVs. A-C negative control with PBS; D-F Plasma samples. A, D): scatter plots; B, E): dot plots with gating for CFSE fluorescence; C, F): dot plots for experimental sample (scatter+ CFSE + ANTI- CD14 antibody); D: dot plots for scatter+ ANTI- CD14 antibody.


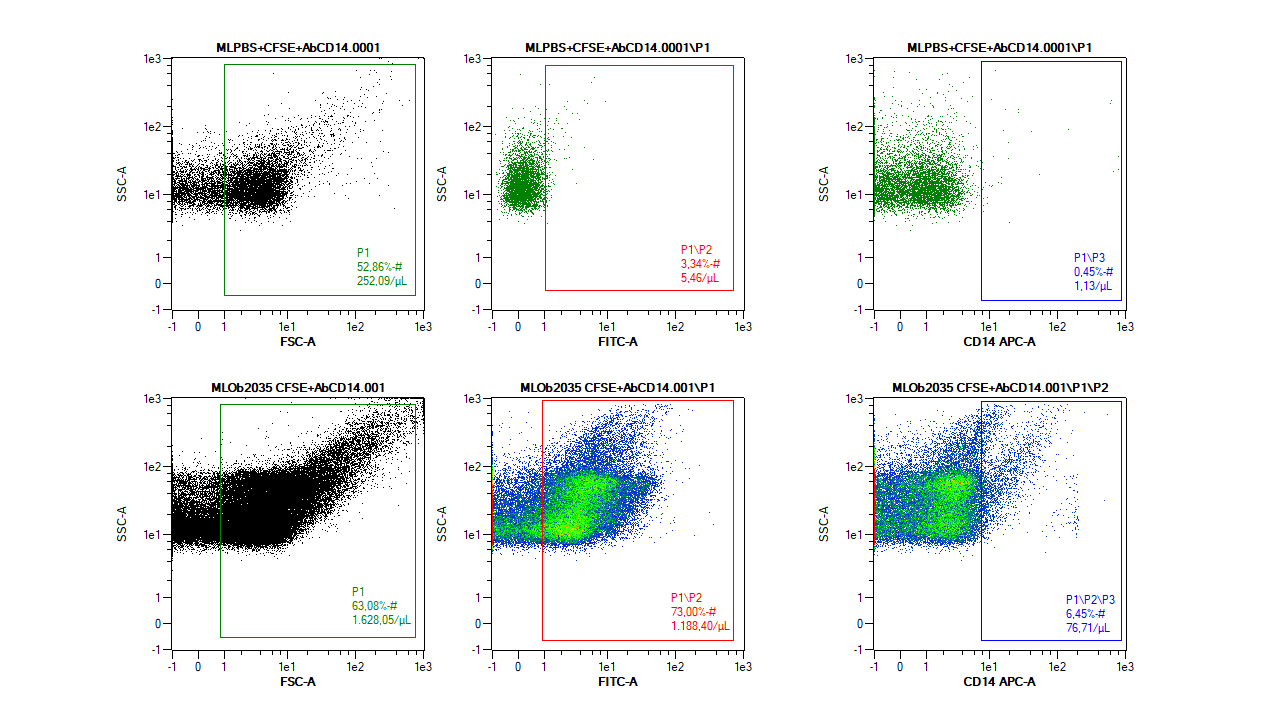


**Supplementary Figure S4:** CD61 gating protocols for plasmatic EVs. A-C negative control with PBS; D-F Plasma samples. A, D): scatter plots; B, E): dot plots with gating for CFSE fluorescence; C, F): dot plots for experimental sample (scatter+ CFSE + ANTI- CD61 antibody); D: dot plots for scatter+ ANTI- CD61 antibody.


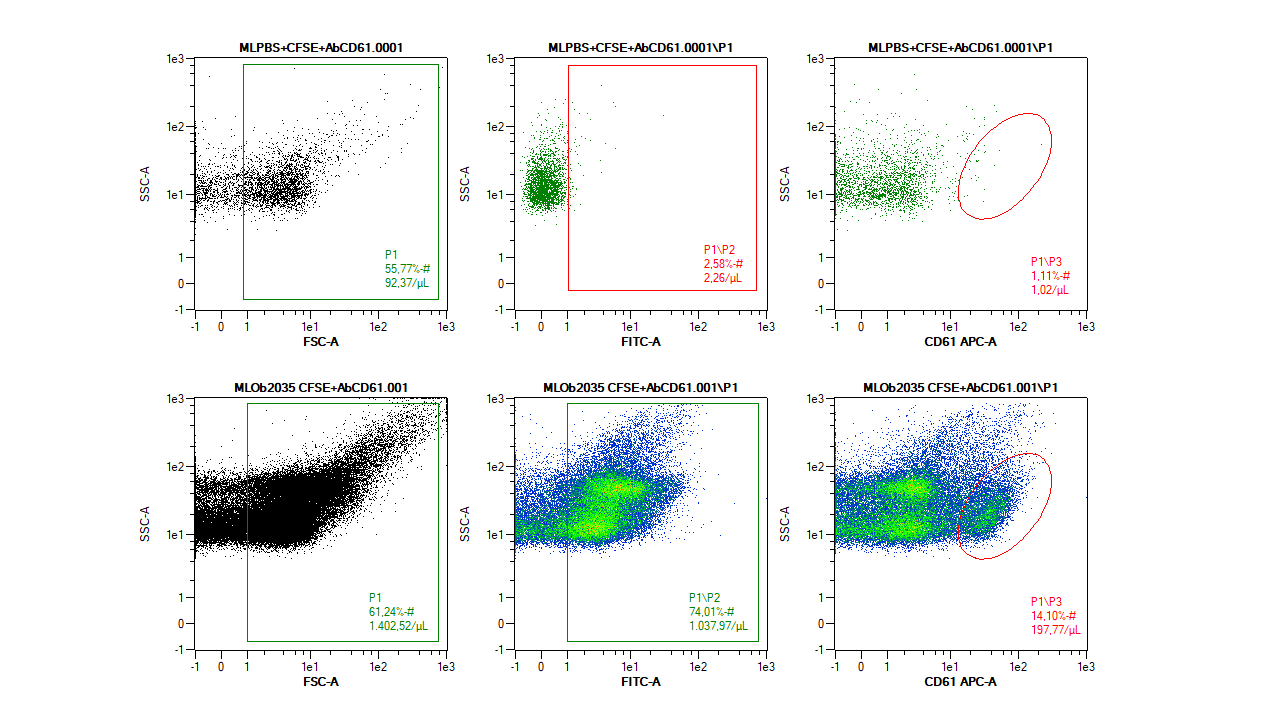


**Supplementary Figure S5:** CD105 gating protocols for plasmatic EVs. A-C negative control with PBS; D-F Plasma samples. A, D): scatter plots; B, E): dot plots with gating for CFSE fluorescence; C, F): dot plots for experimental sample (scatter+ CFSE + ANTI- 105 antibody); D: dot plots for scatter+ ANTI- 105 antibody.


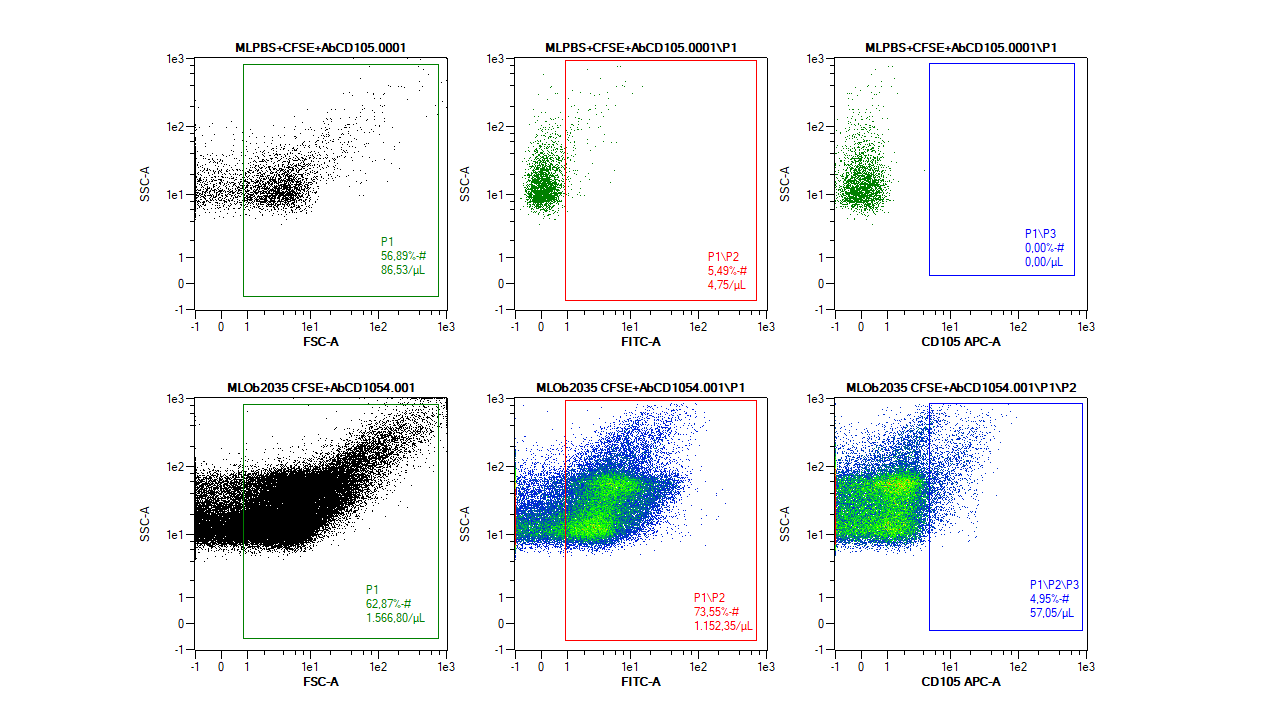


**Supplementary Figure S6:** CD61 gating protocols for plasmatic EVs. A-C negative control with PBS; D-F cell culture media. A, D): scatter plots; B, E): dot plots with gating for CFSE fluorescence; C, F): dot plots for experimental sample (scatter+ CFSE + ANTI- CD66 antibody); D: dot plots for scatter+ ANTI- CD66 antibody.


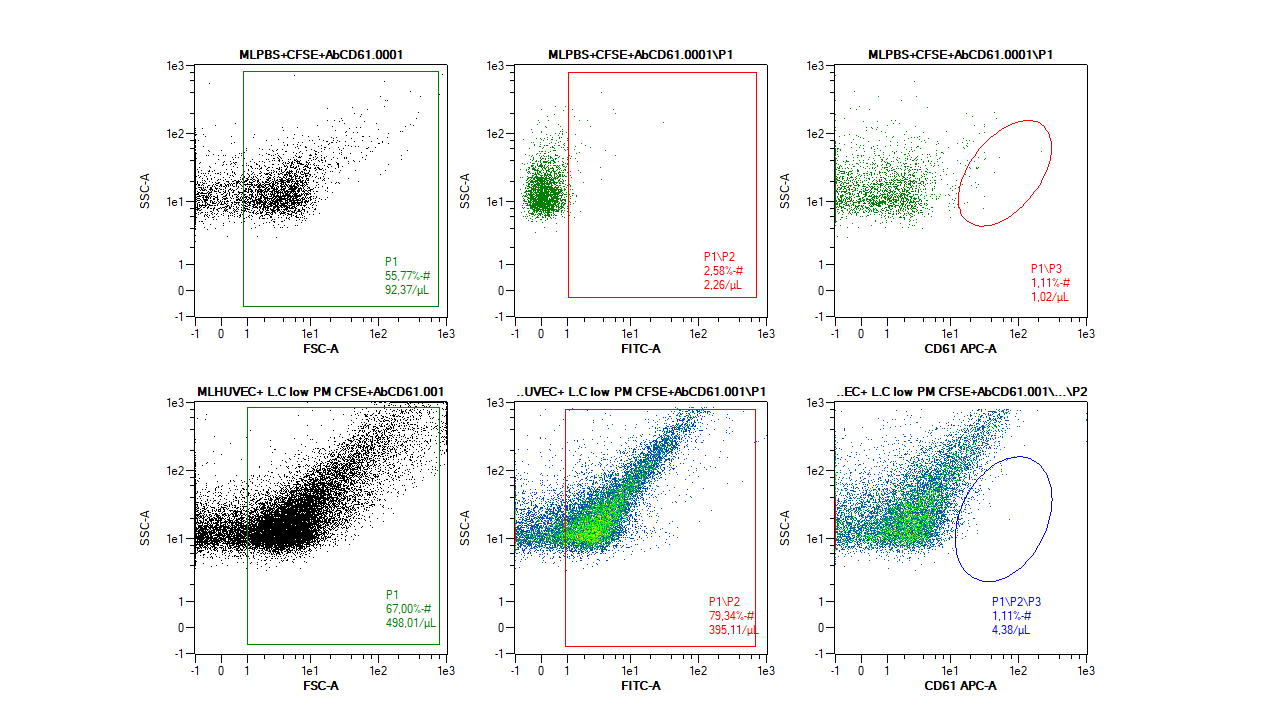


**Supplementary Figure S7:** CD62e gating protocols for primary endothelial cells EVs. A-C negative control with PBS; D-F cell culture media. A, D): scatter plots; B, E): dot plots with gating for CFSE fluorescence; C, F): dot plots for experimental sample (scatter+ CFSE + ANTI- CD62e antibody); D: dot plots for scatter+ ANTI- CD62e antibody.

**
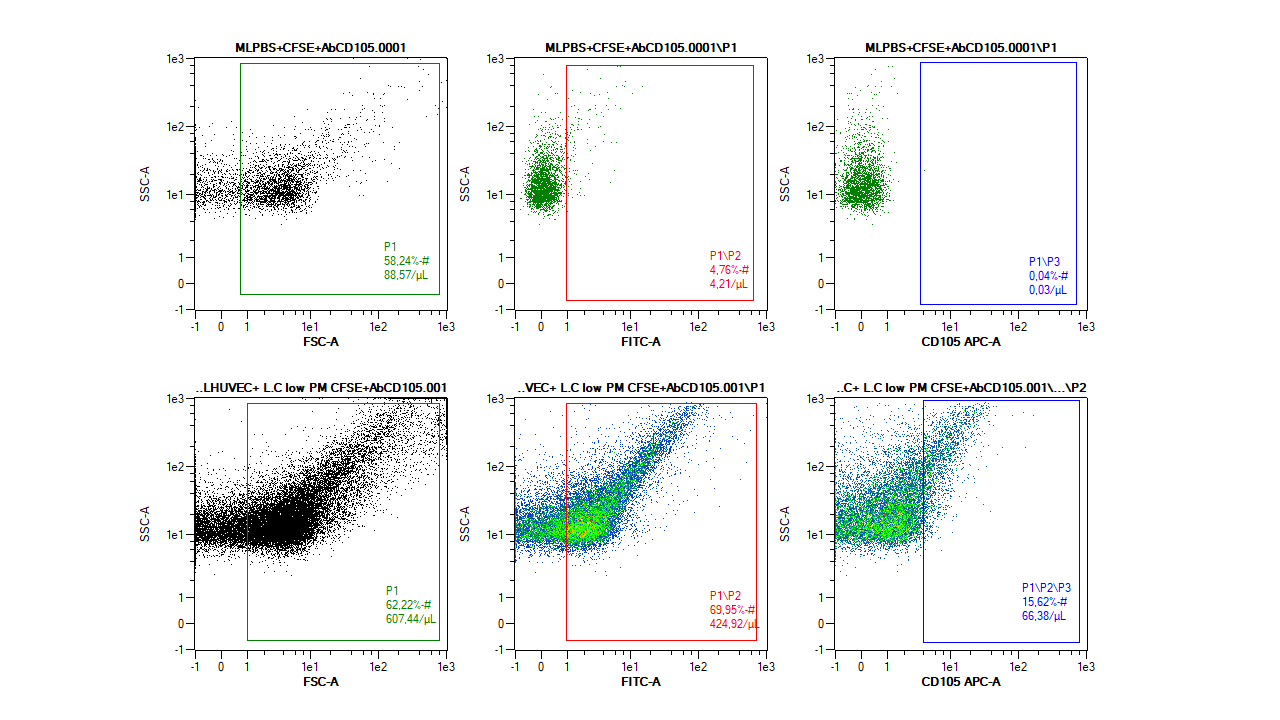
**

**Supplementary Figure S8:** CD105 gating protocols for primary endothelial cells EVs. A-C negative control with PBS; D-F cell culture media. A, D): scatter plots; B, E): dot plots with gating for CFSE fluorescence; C, F): dot plots for experimental sample (scatter+ CFSE + ANTI- CD105 antibody); D: dot plots for scatter+ ANTI- CD105 antibody.


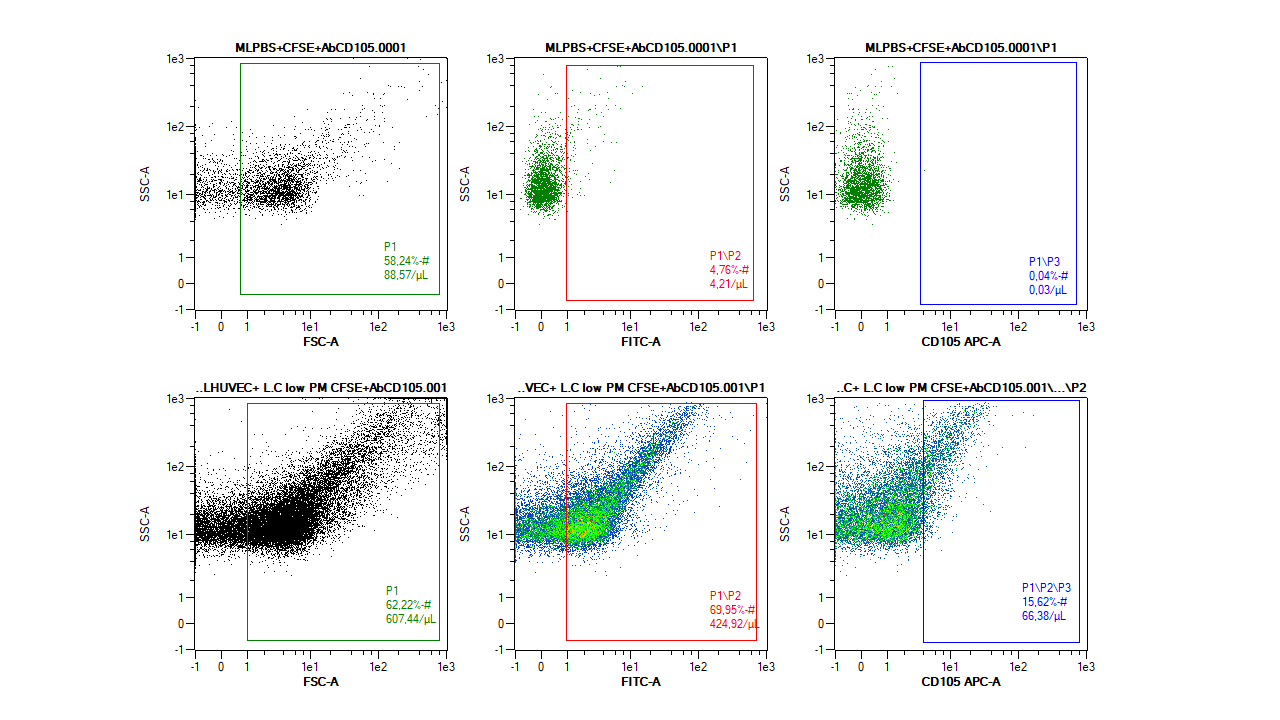


**Supplementary Figure S9** vitality assay (cells count/µL) after treatments of HUVEC cells with OW EVs and NW EVs at both Low and High PM exposures.
